# Patient reported outcomes based on EQ-5D-5L questionnaires in head and neck cancer patients: a real-world study

**DOI:** 10.1186/s12885-022-10346-4

**Published:** 2022-11-29

**Authors:** Tanja Sprave, Eleni Gkika, Vivek Verma, Anca-Ligia Grosu, Raluca Stoian

**Affiliations:** 1 Department of Radiation Oncology, Freiburg University Medical Center, Robert-Koch-Str. 3, 79106 Freiburg, Germany; 2 German Cancer Consortium (DKTK) Partner Site Freiburg, German Cancer Research Centre (DKFZ), Heidelberg, Germany; 3 Department of Radiation Oncology, University of Texas M.D. Anderson Cancer Center, Houston, TX USA

**Keywords:** Head-and-neck cancer, Patient reported outcome, Radiotherapy, EQ-5D-5L

## Abstract

**Objective:**

Health economic comparisons of various therapies are often based on health-related quality of life (HRQOL) using EQ-5D questionnaires within the framework of clinical trials. This real-world study prospectively evaluates the patient reported outcomes (PROs)-based HRQOL of head-and-neck (H&N) cancer patients undergoing modern radiotherapy (RT) to reflect PRO trajectories.

**Methods:**

All H&N cancer patients treated in our clinic between July 2019 and December 2020 who completed the self-reported validated EQ-5D-5L questionnaire (health state index (HI) and Visual Analog Scale (VAS)) at baseline, end of radiotherapy, and at each respective follow up (FU) were included. Descriptive analysis of clinical and sociodemographic data, the frequency and level of each dimension was conducted. To assess the significance of therapy-induced HRQOL changes within and between the group, a distribution-based approach was used.

**Results:**

Altogether, 366 participants completed a total of 565 questionnaires. For the whole cohort, HI at baseline was 0.804 (±0.208), 0.830 (±0.162) at RT completion, 0.812 (±0.205) at the first follow-up, and 0.769 (±0.224) at the second follow-up. The respective VAS values were 62.06 (±23,94), 66.73 (±82.20), 63.30 (±22.74), and 65.48 (±23.39). Females showed significantly lower HI values compared to males, but only at baseline (*p =* 0.034). Significantly lower HI values were also seen in patients with definitive RT as compared to adjuvant RT at baseline (*p =* 0.023), the second follow-up (*p =* 0.047), and the third follow-up (*p =* 0.010). As compared to outpatients, inpatients had significantly lower HI values at RT completion (*p =* 0.017), the second follow-up (*p =* 0.007), and the third follow-up (*p =* 0.031). Subgroup analyses by age (< 65 vs. ≥65) and smoking status (smokers vs. non-smokers) showed no difference at any time point.

**Conclusion:**

PROs demonstrated detectability of time- and intra−/inter-group therapy-induced HRQOL changes. A further detailed exploration of EQ-5D-5L responsiveness for H&N cancer patients is required.

**Supplementary Information:**

The online version contains supplementary material available at 10.1186/s12885-022-10346-4.

## Introduction

Patient-reported outcomes (PROs) refer to a generic manner of measuring subjectively perceived health status [1] that is self-reported by the patient [2, 3]. For economic health-technology-assessment (HTA) evaluations, the generic EQ-5D is the most commonly recommended multi-attribute utility instrument in pharmacoeconomic guidelines internationally [4, 5]. Currently, there is no agreement on the construction and use of health utility data for HTA. The recommendations are unclear regarding who should complete the questionnaire (patient vs physician) as well as how many measurement time points are needed to reflect treatment effects [6]. The adjustment of economic models to baseline, comorbidity, or age is also not standardized [6]. However, PROs provide independent prognostic information for overall survival at various stages of cancer disease [7].

Of note, in recent years, there has been a worldwide shift from EQ-5D-3L to EQ-5D-5L for more accurate measurement of health conditions [8]. Unexpectedly, the current country-specific comparisons between 3 L and 5 L tariffs show that these tariffs are not interchangeable [9], which lead to substantial differences with unpredictable directional results. As a consequence, in most cases the application of 5 L leads to the incremental quality-adjusted-life-years (QALYs) gain appearing to be lower [9]. Accordingly, the EQ. 5D-5L application may lead to significant changes in cost-effectiveness estimates compared to 3 L [9]. The country-specific effects for the health care system have yet to be evaluated [10, 11].

Above all, the substantial QOL impairment caused by diagnosis and therapy of head and neck (H&N) cancer is being intensively investigated [12]. For this purpose, the disease-specific and site-specific QOL assessments are often used [13–15]. Multiple determinants of QOL [16, 17] have been identified and effective interventions have been implemented in routine clinical practice [12]. In light of this, herein, PROs were assessed using validated EQ-5D-5L [18] questionnaires in (H&N) cancer patients undergoing modern radiotherapy (RT) [19]. This real-world analysis outside of clinical trials [20] should theoretically capture the changes in HRQOL over time and provide an updated dataset for further economic evaluation and support the decisions in HTA.

## Methods

All patients who were seen pre-therapeutically and/or post-therapeutically during follow-up for H&N cancers between July 2019 and December 2020 at the University of Freiburg Medical Center and completed questionnaires were included in this analysis. The questionnaires were administered at the initial consultation, at the end of radiotherapy, and at the respective follow-ups [21]. Acute toxicities were retrospectively assessed at the end of RT according to the National Cancer Institute’s Common Terminology Criteria for Adverse Events (CTCAE v5.0). HRQOL was recorded as follows: first FU at 3 months, second FU at 6 months, third FU at 12 months, fourth FU at 24 months, fifth FU at 36-60 months.

The EQ-5D-5L is a generic quantitative measure for the generated health state index score (HI) from the societal perspective and subjective perceived health. The first descriptive element refers to HI, as related to the five dimensions of mobility, self-care, daily activities, pain, and anxiety. There are five possible grades for each variable (no, slight, moderate, severe and extreme problems/inability). The second part provides a visual analogue scale (VAS) for individually perceived health from 0 to 100 (corresponding to the worst to the best imaginable health).

Treatment concepts for each patient were based on multidisciplinary tumor board recommendations. Briefly, definitive approach was recommended for locally advanced and inoperable tumors. Adjuvant approach were based on operative pathologic results. Tumor nomenclature was performed according to the 8th Edition of the UICC TNM Classification of Malignant Tumors. The elective lymphatic nodes and low-risk mucosa were treated to a total dose of 50–54 Gy in five daily fractions of 1.8–2.0 Gy. Macroscopic lymph nodes received 66–70 Gy using a simultaneous boost concept. After surgical resection, the tumor and/or lymph node bed was treated to a total dose of 60–66 Gy depending on pathologic findings.

Demographic and treatment characteristics were obtained from the electronic patient records. Participants with a smoking history of at least 10 pack years were considered as smokers.

Descriptive analysis of clinical and sociodemographic data was conducted with IBM SPSS Statistics software version 25 (IBM, Armonk, NY, USA). Descriptive statistics of the mean values of the EQ-5D-5L HI and VAS values was done. Normal distributions were tested using the Shapiro-Wilk test. Since the tested variables showed the absence of a Gaussian distribution, non-parametric tests were performed. Stratifications were made for two RT regimens (definitive vs. adjuvant radiotherapy), concomitant chemotherapy vs. no chemotherapy, age groups (< 65 vs. ≥65 years), inpatient vs. outpatient status, male vs. female, as well as patients with smoker vs. non-smoker history. A two-tailed *p*-value of < 0.05 was considered statistically significant for all analyses (α = 0.05). Descriptive statistics for five EQ-5D dimensions was done with Excel software version 10.0 (Microsoft Corporation Software, USA).

## Results

### Patient Population and Treatment

**Fig. 1:**
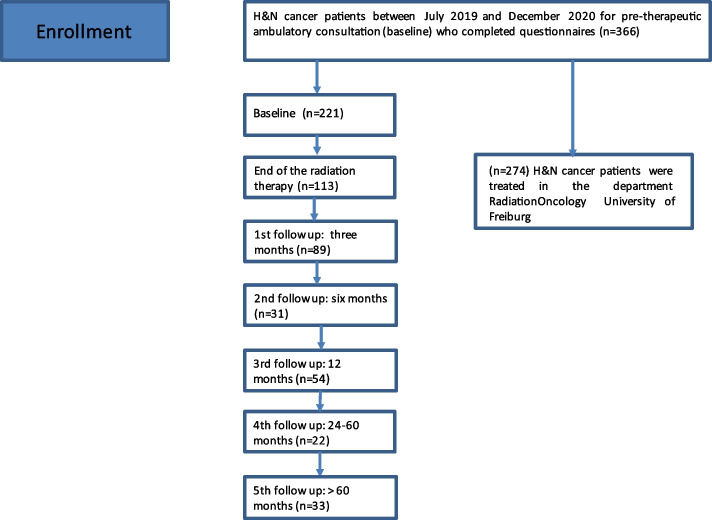
Flow chart

In total, 366 H&N cancer patients completed the questionnaires, yielding 565 total questionnaires (Fig. 1). Baseline characteristics are shown in Table 1. This study included predominantly male patients (*n =* 256, 69.9%) with a median Charlson comorbidity index of 4 (range 2-13) points. Cardiovascular comorbidity was the most common (*n =* 109, 39.8%). The median age was 64 (range 26-96) years. The most common H&N site was in the oropharynx (*n =* 138, 37.7%) The most frequent histology was squamous cell carcinoma (*n =* 279, 76.2%). Overall, 216 (59%) patients received definitive treatment and 150 (41.0%) patients received adjuvant treatment (Table 1). Chemotherapy was given for 173 (47.3%) cases. Eighteen (4.9%) patients underwent re-irradiation for unresectable recurrent tumors. The vast majority of patients (*n =* 250, 91.2%) received intensity modulated RT (IMRT). In total, 191 (52.2%) patients required an inpatient admission for a median of 23 (range 1-55) days.

**Table 1:** Baseline characteristics of the study population (*n =* 366). Comorbidities were collected only for the patients with complete medical histories (*n =* 274)

	n (%)
***All (n = 366)***	
**Age** (median, range)	64 (26-96)
**Insurance**	
private	16 (4.4)
statutory	350 (95.6)
**Sex**	
female	110 (30.1)
male	256 (69.9)
**Smoking**	
no	128 (35.0)
yes	150 (41.0)
unknown	88 (24.0)
**Localisation**	
nasopharynx	15 (4.1)
oropharynx	138 (37.7)
hypopharynx	42 (11.5)
larynx	51 (13.9)
oral cavity	51 (13.9)
salivary glands	19 (5.2)
paranasal sinus	17 (4.6)
others	33 (9.0)
**Histology**	
squamous cell carcinoma	279 (76.2)
adenocarcinoma	35 (9.6)
undifferentiated	24 (6.6)
others	28 (7.7)
**Grading**	
1	31 (8.5)
2	185 (50.5)
3	110 (30.1)
4	24 (6.6)
unknown	16 (4.4)
**Radiotherapy**	
definitive	216 (59.0)
adjuvant	150 (41.0)
re-radiation	18 (4.9)
**Radiotherapy technique** ***(n = 274)***	
3D	24 (8.8)
IMRT	250 (91.2)
**Concomitant chemotherapy**	
yes	173 (47.3)
no	180 (49.2)
unknown	13 (3.6)
**Inpatient, days** (median, range)	23 (1-55)
yes	191 (52.2)
no	171 (46.7)
unknown	4 (1.1)
**Secondary malignancy**	
yes	36 (9.8)
**Charlson score**	
(median, range) at the baseline	4 (2-13)
**Comorbidity** ***(n = 274)***	
Neurological comorbidity	24 (8.8)
Nephrological comorbidity	14 (5.1)
Cardiovascular comorbidity	109 (39.8)
Diabetes mellitus	27 (9.9)
COPD	20 (5.5)
One comorbidity	78 (28.5)
Two comorbidities	29 (10.6)
Three comorbidities	9 (3.3)
Four comorbidities	5 (1.8)
Five comorbidities	1 (0.4)

*Abbreviation*: *COPD* Chronic obstructive pulmonary disease, *IMRT* Intensity modulated radiotherapy, others: carcinoma of unknown primary; thyroid gland carcinoma

In summary, the rates of grade 3 treatment-related toxicities were low, and included dysgeusia (*n =* 55), dysphagia (*n =* 50), and oral mucositis (*n =* 46) (Table 2). There were no grade ≥ 4 toxicities.

**Table 2:** Radiotherapy-related toxicities according at the end of radiotherapy to the Common Terminology Criteria for Adverse Events (CTCAE v5.0)

***All n = 274***				
CTCAE grade	0	1	2	3
Dermatitis	59	102	85	27
Dysphagia	77	73	73	50
Dysgeusia	132	48	38	55
Nausea	244	26	3	0
Mucositis	75	59	93	46
Xerostomia	168	90	14	1
Pain	132	72	35	34
Hoarseness	238	30	3	2
Dyspneu	264	5	4	0

*Abbreviation*: *CTCAE* Common Terminology Criteria for Adverse Event

### HI and VAS

For the whole cohort, the mean EQ-5D-5L HI before treatment, after treatment, and at the first, second, third, fourth, and fifth FU was 0.804 (standard deviation, SD 0.21), 0.830 (SD 0.16), 0.812 (SD 0.2), 0.769 (SD 0.22), 0.834 (SD 0.22), 0.840 (SD 0.19), and 0.877 (SD 0.13), respectively (*p* > 0.05 for all) (Table 3). The corresponding mean VAS score for the whole group before treatment, after treatment, and at the first, second, third, fourth, and fifth FU was 62.06 (SD 23.94), 66.73 (SD 82.2), 63.3 (SD 22.74), 65.48 (SD 23.39), 77.30 (SD 19.92, *p <* 0.01), 67.27 (SD 19.07), and 73.64 (SD 15.48, *p <* 0.01), respectively (*p* > 0.05 for all) (Table 3).

**Table 3:** EQ-5D-5L health index and EQ-VAS values for whole study population (*n =* 366) at the baseline, end of treatment, first, second, third, fourth and fifth follow up

***All n = 366***	Health utility index						
All ***n =*** 366	Questionnaries ***n =*** 565	Mean	SD	SE of diff	Mean diff	0.5 SD	***p-***value^**a**^
Baseline	221	0,804	0,208			0,104	
RT end	114	0,830	0,162	0,023	−0,026	0,081	0,927
1 FU	89	0,812	0,205	0,028	0,018	0,103	0,802
2 FU	32	0,769	0,224	0,041	0,042	0,112	0,302
3 FU	54	0,834	0,217	0,044	−0,064	0,109	0,087
4 FU	22	0,840	0,192	0,050	−0,006	0,100	0,555
5 FU	33	0,877	0,127	0,054	-0,037	0,064	0,058
***All n = 366***	**VAS**						
**All** ***n =*** **366**	**Questionnaries** ***n =*** **565**	**Mean**	**SD**	**SE of diff**	**Mean diff**	**0.5 SD**	***p-*value**^**a**^
Baseline	221	62,06	23,94			11,97	
RT end	114	66,73	82,20	4,87	−4,66	41,10	0,266
1 FU	89	63,30	22,74	5,29	−1,24	11,37	0,711
2 FU	32	65,48	23,39	8,07	−3,42	11,70	0,526
3 FU	54	77,30	19,92	6,39	−15,23	9,96	**< 0,0001**
4 FU	22	67,27	19,07	9,41	−5,21	9,54	0,416
5 FU	33	73,64	15,48	7,85	−11,57	7,74	**0,009**

*Abbreviation*: ^a^Mann-Whitney U test for comparison of health utility values at the end of radiation and follow up each to baseline, *RT* Radiotherapy, *SD* Standard deviation, *SE of diff* Standard error of difference, *VAS* Visual analog scale

Interestingly, the subgroup comparison of HI between those aged < 65 vs ≥65 years, both within each group and between groups, did not show significant differences at any time of the assessment (*p* > 0.05 for all) (Table 4, Supplemental Table 1). In the gender-specific analysis, women reported significantly lower HI values (0.765, SD 0.23) in contrast to their male counterparts (0.822, SD 0.2) but only at baseline (*p =* 0.034; p > 0.05 for the remainder) (Table 4, Supplemental Table 1). The same tendency for HI without any meaningful difference (p > 0.05 for all) was seen in the comparison between smokers and non-smokers (Table 4, Supplemental Table 1).

**Table 4:** EQ-5D-5L health index in H&N cancer patients at the baseline and end of treatment for the following subgroups: < 65 vs. ≥65 years old, male vs. female, definitive vs. adjuvant radiotherapy approach, smoker vs. non-smoker, inpatients vs. outpatients, concomitant chemotherapy vs. no chemotherapy

**<65y vs. ≥65y health index values**									
	Questionnaires (n)	Mean of <65y	SD	*p-*value^a^	Questionnaires (n)	Mean of ≥65y	SD	*p-*value^a^	*p-*value^b^
Baseline	105	0,823	0,146	0,871	117	0,782	0,229	0,277	0,976
RT end	54	0,830	0,179		59	0,837	0,177		0,885
Total	159				176				
**Male vs. female health index values**									
	Questionnaires (n)	Mean of male	SD	*p-*value^a^	Questionnaires (n)	Mean of female	SD	*p-*value^a^	*p-*value^b^
Baseline	153	0,822	0,196	0,541	68	0,765	0,231	0,118	**0,034**
RT end	77	0,837	0,150		36	0,816	0,188		0,561
Total	230				104				
**Definitive vs. adjvant radiotherapy**									
	Questionnaires (n)	Mean of definitive	SD	*p-*value^a^	Questionnaires (n)	Mean of adjuvant	SD	*p-*value^a^	*p-*value^b^
Baseline	138	0,777	0,223	0,092	83	0,849	0,173	0,193	**0,023**
RT end	64	0,825	0,152		49	0,837	0,177		0,429
Total	202				132				
**Smoker vs. non-smoker**									
	Questionnaires (n)	Mean of smoker	SD	*p-*value^a^	Questionnaires (n)	Mean of non-smoker	SD	*p-*value^a^	*p-*value^b^
Baseline	76	0,817	0,177	0,080	67	0,832	0,212	0,806	0,116
RT end	60	0,833	0,162		42	0,836	0,169		0,779
Total	136				109				
**Inpatient vs.outpatient**									
	Questionnaires (n)	Mean of inpatient	SD	*p-*value^a^	Questionnaires (n)	Mean of outpatient	SD	*p-*value^a^	*p-*value^b^
Baseline	106	0,812	0,201	**0,048**	115	0,797	0,216	0,176	0,941
RT end	71	0,806	0,164		42	0,870	0,153		**0,017**
Total	177				157				
**Chemotherapy vs.no chemotherapy**									
	Questionnaires (n)	Mean of chemotherapy	SD	*p-*value^a^	Questionnaires (n)	Mean of no chemotherapy	SD	*p-*value^a^	*p-*value^b^
Baseline	92	0,870	0,134	**0,021**	117	0,764	0,239	**0,031**	0,012
RT end	51	0,831	0,144		60	0,832	0,179		0,624
Total	143				177				

*Abbreviation*: ^a^within group, *p-*value Kruskal-Wallis test; ^b^between groups *p*-value: Mann-Whitney U test, *FU* Follow up, *RT* Radiotherapy, *SD* Standard deviation, *VAS* Visual analog scale

The sole analysis of cohorts according to therapy approaches was as follows. Definitive vs. adjuvant radiotherapy showed no significant changes in HI scores within groups (definitive: *p =* 0.092, adjuvant: *p =* 0.193) (Table 4, Supplemental Table 1). When comparing between groups, patients with definitive intent consistently showed significantly lower HI values in contrast to the adjuvant cohort at baseline (0.777 (SD 0.22) vs. 0.849 (SD 0.17), *p =* 0.023), at the second FU (0.734 (SD 0.17) vs. 0.789 (SD 0.25), *p =* 0.047), and at the third FU (0.752 (SD 0.26) vs. 0.928 (SD 0.08), *p =* 0.010) (Table 4, Supplemental Table 1). In the inter-group comparison, patients in the RT alone group had significantly reduced HI values compared to the chemoradiotherapy (CRT) cohort at baseline (0.764 (SD 0.24) vs. 0.870 (SD 0.13), *p =* 0.012) (Table 4). At the end of therapy, there was no difference between the CRT and RT groups (*p =* 0.624) (Table 4).

The analysis of patients who had an inpatient admission during RT alone demonstrated significant HI changes during the observation period (*p =* 0.048) (Table 4, Supplemental Table 1). In contrast, the group of patients without inpatient admission did not experience any significant HI changes within the group (*p =* 0.176) (Table 4, Supplemental Table 1). The inter-group comparison revealed a significant difference in consistently lower HI values in the inpatient cohort at completion of RT (0.806 (SD 0.16) vs. outpatient 0.870 (SD 0.15), *p =* 0.017), at the second FU (0.712 (SD 0.24) vs. 0.875 (SD 0.14), *p =* 0.007), and the third FU (0.784 (0.24) vs. 0.901 (SD 0.15), *p =* 0.031) (Table 4, Supplemental Table 1).

The subgroup analysis of the EQ-VAS within groups demonstrated a significant change over the observation period in those < 65 years (*p <* 0.0001), but not in those ≥65 years (*p =* 0.15) (Table 5). Remarkably, there was a significant difference between only EQ-VAS scores at the end of RT (< 65 years 54.63 (SD 20.71) vs. ≥65 years 77.80 (SD 21.3), *p =* 0.009) (Table 5, Supplemental Table 2).

**Table 5:** Corresponding EQ-VAS values for aforementioned subgroups

**<65y vs. ≥65y health index values**									
	Questionnaires (n)	Mean of <65y	SD	*p-*value^a^	Questionnaires (n)	Mean of ≥65y	SD	*p-*value^a^	*p-*value^b^
Baseline	105	60,38	24,01	**< 0.0001**	117	63,58	23,87	0,155	0,343
RT end	54	54,63	20,71		59	77,80	21,30		**0,009**
Total	159				176				
**Male vs. female health index values**									
	Questionnaires (n)	Mean of male	SD	*p-*value^a^	Questionnaires (n)	Mean of female	SD	*p-*value^a^	*p-*value^b^
Baseline	153	63,34	24,12	**0,001**	68	59,18	23,43	0,057	0,142
RT end	77	70,78	28,45		36	58,06	21,49		0,655
Total	230				104				
**Definitive vs. adjvant radiotherapy**									
	Questionnaires (n)	Mean of definitive	SD	*p-*value^a^	Questionnaires (n)	Mean of adjuvant	SD	*p-*value^a^	*p-*value^b^
Baseline	138	61,26	24,42	**0,010**	83	63,41	23,19	**0,0004**	0,598
RT end	64	71,88	27,80		49	60,00	20,46		0,959
Total	202				132				
**Smoker vs. non-smoker**									
	Questionnaires (n)	Mean of smoker	SD	*p-*value^a^	Questionnaires (n)	Mean of non-smoker	SD	*p-*value^a^	*p-*value^b^
Baseline	76	63,16	23,34	**0,0005**	67	65,33	22,21	0,252	0,637
RT end	60	56,83	21,41		42	63,57	19,04		0,187
Total	136				109				
**Inpatient vs.outpatient**									
	Questionnaires (n)	Mean of inpatient	SD	*p-*value^a^	Questionnaires (n)	Mean of outpatient	SD	*p-*value^a^	*p-*value^b^
Baseline	106	63,13	23,36	**0,0043**	115	61,06	24,53	**0,0007**	0,452
RT end	71	68,10	12,80		42	64,40	20,49		**0,048**
Total	177				157				
**Chemotherapy vs.no chemotherapy**									
	Questionnaires (n)	Mean of chemotherapy	SD	*p-*value^a^	Questionnaires (n)	Mean of no chemotherapy	SD	*p-*value^a^	*p-*value^b^
Baseline	92	66,07	22,12	0,016	117	59,74	24,79	**0,031**	0,065
RT end	51	72,55	12,7		60	62,83	19,99		0,109
Total	143				177				

*Abbreviation*: ^a^within group, *p*-value Kruskal-Wallis test; ^b^between groups p-value: Mann-Whitney U test, *FU* Follow up, *RT* Radiotherapy, *SD* Standard deviation, *VAS* Visual analog scale

Gender-specific analysis revealed a significant change in EQ-VAS for men within the group (*p =* 0.001), but not for women (*p =* 0.057). The gender inter-group comparison showed no difference in the observation period (Table 5, Supplemental Table 2).

Both the cohorts with definitive and adjuvant RT showed significant EQ-VAS changes within the groups (definitive: *p =* 0.010, adjuvant: *p =* 0.0004) (Table 5, Supplemental Table 1). Comparison between these groups only at the first FU showed significantly lower EQ-VAS scores in the definitive group (59.30 (SD 22.35) vs. adjuvant 70.44 (SD 21.99), i0.013) (Table 5, Supplemental Table 1). CRT vs. all RT groups showed significant changes in EQ-VAS scores within groups (CRT: *p =* 0.016, RT: *p =* 0.031) (Table 5). At the end of therapy, there was no difference in EQ-VAS between the CRT and RT group (*p =* 0.109) (Table 5, Supplemental Table 2).

Smokers showed a significant change in EQ-VAS scores during the observation period (*p =* 0.0005), but not non-smokers (*p =* 0.252) (Table 4, Supplemental Table 1). The inter-group comparison between smokers and non-smokers discerned no difference at any time point (*p* > 0.05 for all) (Table 5, Supplemental Table 2).

Patients with inpatient stay and lack thereof showed significant intra-group EQ-VAS changes (inpatient: *p =* 0.0043, outpatient: *p =* 0.0007) (Table 5).

**Fig. 2:**
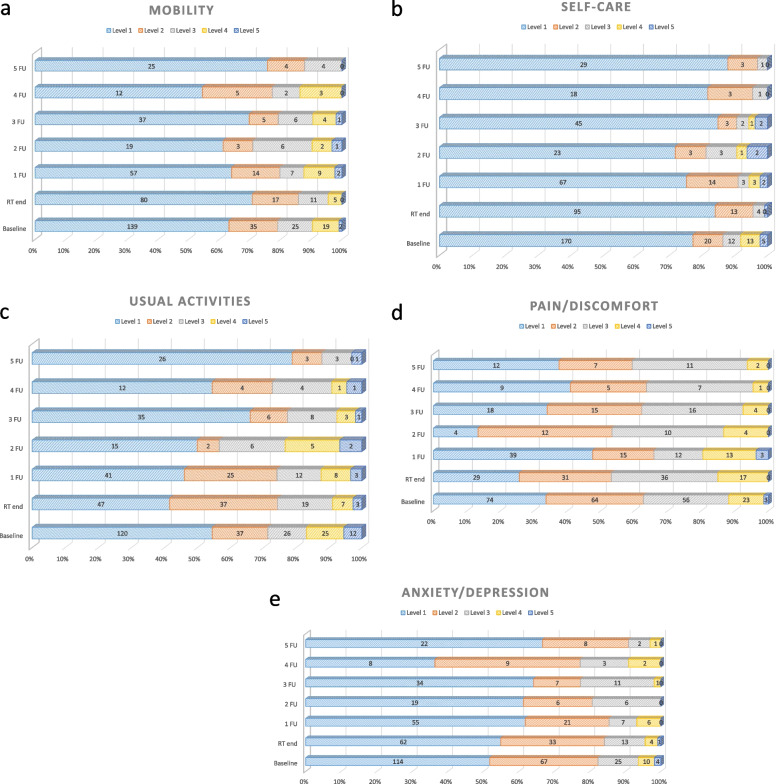
a-e: EQ-5D-5L frequencies and proportions reported by dimension and level for all H&N cancer patients (*n =* 366): **a** Mobility, **b** self-care, and **c** usual activities: 1-no problems, 2-slight problems, 3-moderate problems, 4-severe problems, 5-unable to; **d** Pain/discomfort: 1-no pain, 2-slight pain, 3-moderate pain, 4-severe pain, 5-extreme pain; **e** Anxiety/depression: 1-not anxious/depressed, 2-slightly anxious/depressed, 3-moderately anxious/depressed, 4-severly anxious/depressed, 5-extremely anxious/depressed

The frequencies of the response patterns for all five dimensions grouped according to pre-therapeutic (baseline) and post-therapeutic (end of treatment) observations and respective follow-up intervals are presented in Table 5 and Fig. 2a-e. All distributions except for pain/discomfort were skewed, with high abundances for the category of “no problems”. The largest number of most severe impairments (level 5) was reported in the “usual activity” category (Table 6, Fig. 2a-e). Most patients experienced anxiety/depression followed by pain/discomfort.

**Table 6:** EQ-5D-5L frequencies and proportions reported by dimension and level for all H&N cancer patients (*n =* 366). Mobility, self-care, and usual activities: 1-no problems, 2-slight problems, 3-moderate problems, 4-severe problems, 5-unable to. Pain/discomfort: 1-no pain, 2-slight pain, 3-moderate pain, 4-severe pain, 5-extreme pain. Anxiety/depression: 1-not anxious/depressed, 2-slightly anxious/depressed, 3-moderately anxious/depressed, 4-severly anxious/depressed, 5-extremely anxious/depressed

Dimension	Baseline	RT end	1 FU	2 FU	3 FU	4 FU	5 FU
**Level**	***n (%)***	***n (%)***	***n (%)***	***n (%)***	***n (%)***	***n (%)***	***n (%)***
***Mobility***							
1	139 (63.2)	80 (70.8)	57 (64.0)	19 (61.3)	37 (69.8)	12 (54.5)	25 (75.8)
2	35 (15.9)	17 (15.0)	14 (15.7)	3 (9.7)	5 (9.4)	5 (22.7)	4 (12.1)
3	25 (11.4)	11 (9.7)	7 (7.9)	6 (19.4)	6 (11.3)	2 (9.1)	4 (12.1)
4	19 (8.6)	5 (4.4)	9 (10.1)	2 (6.5)	4 (7.5)	3 (13.8)	0
5	2 (0.9)	0	2 (2.2)	1 (3.2)	1 (1.9)	0	0
***Self-care***							
1	170 (77.3)	95 (84.1)	67 (75.3)	23 (74.2)	45 (84.9)	18 (81.8)	29 (87.9)
2	20 (9.1)	13 (11.5)	14 (15.7)	3 (9.7)	3 (5.7)	3 (13.6)	3 (9.1)
3	12 (5.5)	4 (3.5)	3 (3.4)	3 (6.5)	2 (3.8)	1 (4.5)	1 (3.0)
4	13 (5.9)	0	3 (3.4)	1 (3.2)	1 (1.9)	0	0
5	5 (2.3)	1 (0.9)	2 (2.2)	2 (6.5)	2 (3.8)	0	0
**Usual activities**							
1	120 (54.5)	47 (41.6)	41 (46.1)	15 (48.4)	35 (66.0)	12 (54.5)	26 (78.8)
2	37 (16.8)	37 (32.7)	25 (28.1)	2 (9.7)	6 (11.3)	4 (18.2)	3 (9.1)
3	26 (11.8)	19 (16.8)	12 (13.5)	6 (19.4)	8 (15.1)	4 (18.2)	3 (9.1)
4	25 (11.4)	7 (6.2)	8 (9.0)	5 (16.1)	3 (5.7)	1 (4.5)	0
5	12 (5.5)	3 (2.7)	3 (3.4)	2 (6.5)	1 (1.9)	1 (4.5)	1 (3.0)
***Pain/discomfort***							
1	74 (33.6)	29 (25.7)	39 (43.8)	4 (12.9)	18 (34.0)	9 (40.9)	12 (39.4)
2	64 (29.1)	31 (27.4)	15 (16.9)	12 (38.7)	15 (28.3)	5 (22.7)	7 (21.2)
3	56 (25.5)	36 (31.9)	12 (13.5)	10 (32.3)	16 (30.2)	7 (31.8)	11 (33.3)
4	23 (10.5)	17 (15.0)	13 (14.6)	4 (16.1)	4 (7.5)	1 (4.5)	2 (6.1)
5	3 (1.4)	0	3 (3.4)	0	0	0	0
***Anxiety/depression***							
1	114 (51.9)	62 (54.9)	55 (61.8)	19 (61.3)	34 (64.2)	8 (36.4)	22 (66.7)
2	67 (30.5)	33 (29.2)	21 (23.6)	6 (19.4)	7 (13.2)	9 (40.9)	8 (24.2)
3	25 (11.4)	13 (11.5)	7 (7.9)	6 (19.4)	11 (20.8)	3 13.6)	2 (6.1)
4	10 (4.5)	4 (3.5)	6 (6.7)	0	1 (1.9)	2 (9.1)	1 (3.0)
5	4 (1.8)	1 (0.9)	0	0	0	0	0
*Questionnaires n = 565*							

*Abbreviation*: *FU* Follow up, *RT* Radiotherapy

## Discussion

The objective of this investigation was to assess real-world PROs based on EQ-5D-5L in H&N cancer patients undergoing modern RT. To the best of our knowledge, this is the first study to report EQ-5D-5L results in the tertiary care center setting in Germany. This trial provides EQ-5D-5L based HI and VAS scores trajectories for the total population and various subgroups. These PROs could be used for studies that do not have a control group or without sufficient FU to compare the short- and long-term treatment outcomes. Most importantly, QALYs can be calculated at different therapy time points and compared internationally.

Our results attest to a relatively high and stable trajectory of HRQOL for the whole cohort (Table 3) and are comparable to our previous findings [19]. Remarkably, no substantial worsening (but rather, improvement) of HRQOL in HI by − 0.026 and VAS by − 4.66 was observed at RT completion despite treatment-related side effects (Table 3). These findings reflect the standard application of established RT techniques such as intensity-modulated RT (IMRT) and rigorous management of radiation-related side effects. It is conceivable that these results show only country- and cohort-specific assessment of HRQOL. In fact, the completion of several weeks of radiotherapy tended to show a slight improvement in mobility and a slight decrease in anxiety (Fig. 2a, e), which may have led to an increase in HI and VAS scores at the end of RT (Table 3). In contrast, the De-ESCALaTe HPV study reported significantly lower HI values of 0.606 in the cisplatin arm and 0.565 in the cetuximab arm at RT completion [22]. Patients in the De-ESCALaTe HPV trial were significantly younger, with a median age of 57 (vs. 64 in our population), and better performance status, both likely because that trial encompassed only HPV-related disease. Furthermore, the median length of inpatient stay was 8-10 days [22], which was significantly shorter compared to 23 days in our patients (Table 1). It is possible that the therapy-related side effects were not adequately treated in the outpatient setting despite the chemotherapeutic dose de-escalation in the De-ESCALaTE HPV trial.

Of note, the mean HI score in patients aged < 65 at the end of RT was 0.830 vs. 0.837 in those ≥65 (*p =* 0.885). These findings are in line with our previous published PROs in elderly H&N patients at the end of RT, with HI of 0.843 [19]. Our results are identical to the HI of 0.84 of the ≥65 year old German general population [23]. For comparison, a study of 85+ in Germany found a mean EQ-VAS score of 62.4 (SD 18.8) and a mean EQ-5D-3L index of 0.77 (SD 0.24) [24]. A Dutch study reported mean EQ-5D-3L indices and EQ-VAS of 0.94 and 84 among 65-69 years old patients, and 0.86 and 76 among those ≥85, respectively [25]. Here, it is important to mention the use of the 3 L version in both studies, which represents a considerable bias. Our VAS scores have a comparable range of 59-78.33 and HI of 0.725 − 0.866 (Table 4, Supplemental Table 1). Additionally, Grochtdreis et al. reported German normative values, comprising HI 0.85 for patients 65-74 years old and a value of 0.80 for those ≥75 years old [26]. In summary, we could not find a linear deterioration of HRQOL with increasing age [23], possibly due to an unrepresentative sample size. Furthermore, despite the modifications of EQ-5D-3L version, the ceiling effect cannot be excluded [27–32]. Thus, using the EQ-5D-5L version still yielded a high rate of optimal HRQOL statuses [33]. However, the 5 L version convinced by the superior measurement properties and was recommended for general use as well as for cancer patients [29, 34]. Hence, the real benefit needs to be examined further [35].

Subgroup analysis in our patients with definitive RT showed significantly lower HI at baseline compared to adjuvant RT (Table 4). In contrast to our results, de Almeida et al. reported slightly higher HI values using the standard gamble method for definitive RT alone with 0.91, with addition of chemotherapy CRT 0.88 and comparable HI for adjuvant setting with 0.89 with RT alone and CRT [36]. Corresponding VAS scores were consistently lower compared to our results: definitive RT alone 0.54, CRT 0.48 and adjuvant RT alone 0.59 and CRT 0.53 [36]. However, the study population of de Almeida et al. consisted of predominantly younger women and is not representative of H&N cancer patients [36]. Interestingly, patients in the CRT group showed higher HI and EQ-VAS scores at baseline compared to the RT alone group (Tables 4 and 5). However, it is possible that indications for chemotherapy could be a confounding factor, meaning that potentially the patients more medically “fit” received CRT.

In our cohort, the women showed significantly lower HI values (0.765, SD 0.23) in comparison to men (0.822, SD 0.2) at baseline (*p =* 0.034), but not any remaining time points (Table 4). A multicenter analysis from three European countries showed comparable data; the mean values of all EQ-5D values were higher in men than in women, but in contrast to our collective, decreased with age [37]. Patients with inpatient admission during RT showed a significantly lower HI value as compared to outpatients (Table 4a), which could be explained by the need for supportive therapy for therapy-related side effects and existing morbidity. Interestingly, at RT completion, the same inpatients reported a higher VAS (68.10) compared to outpatients VAS (64.40) (*p =* 0.048). Further exploration using more appropriate instruments is needed to identify and interpret the multifactorial influence.

Despite the already established recommendation for the interpretation of PROs to assess the benefit of an intervention in cancer patients, a continuing challenge in HRQOL remains ascertaining the adequate way to translate the results [38, 39], particularly with regard to defining what constitutes a minimally important difference (MID) or minimal clinical important difference. The MID was defined as the smallest change in a PRO measurement that is perceived by patients as beneficial or that would result in a change in treatment [40]. An MID is typically unique to the population being studied [39], but MID values for H&N cancer patients are not available. For this reason, we only compared the mean values in the evaluation to show the significance of the changes.

The strength of our study is the large representative sample size for H&N cancer patients undergoing RT. Another strength is that all HRQOL data was self-reported and is the illustration of HRQOL changes over time and importantly in comparison to baseline. Additionally, some limitations such as the single-center nature of this study and differing numbers of patients during the observation period must be mentioned.

On the other hand, the small number of patients with heterogenous H&N cancers at various FU times may not have been sufficient to finely discern small potential HI and VAS differences. In particular, the different number of participants at various FU examinations possibly led to biases and needs to be evaluated in larger cohorts. It is possible that the adverse events were not fully collected and comprehensively documented which could result in bias. It is possible that our cohort is not representative of H&N cancer patients and rather represents comorbid patients requiring increased care in the university setting; thus, the generalizability of these results is unclear. Nevertheless, further inter-institutional PRO research should expand these findings and expand the understanding of the PRO trajectory and in particular its relevance for HTA.

## Conclusion

PROs using EQ-5D-5L demonstrated the ability to detect the time-based and inter−/intra- subgroup-specific therapy-induced HRQOL changes. Given the diverse therapeutic approaches in the treatment of H&N cancer patients, as well as time-specific occurrence of side effects that may affect HQOL, multiple PRO assessments for economic evaluation is recommend. A further detailed exploration of EQ-5D-5L responsiveness for H&N cancer patients is required.

## Supplementary Information

**Additional file 1.**

**Additional file 2.**

## Data Availability

The datasets used and analyzed during the current study are available from the corresponding author on reasonable request.

## Abbreviations

CRT: Chemoradiotherapy
CTCAE: Common Terminology Criteria for Adverse Events
IMRT: Intensity modulated radiotherapy
FU: Follow up
H&N: Head-and-neck cancer
HI: Health index state
HRQOL: Health related quality of life
HTA: Health technology assessment
MID: Minimally important difference
PRO: Patient reported outcome
RT: Radiotherapy
QALY: Quality-adjusted life years
VAS: Visual analog scale
Vs: Versus
