# Supplementary material for: Patient reported outcomes based on EQ-5D-5L questionnaires in head and neck cancer patients: a real-world study
Source: BMC Cancer. 2022 Nov 29;22:1236. doi: 10.1186/s12885-022-10346-4 (PMC9710161; doi:10.1186/s12885-022-10346-4)
Supplement: Supplementary file 1 — Additional file 1. [file 12885_2022_10346_MOESM1_ESM.docx]

**Supplement table 1:** EQ-5D-5L health index values in H&N cancer patients at the baseline, the end, the first, second, third, fourth, and fifth follow up for the following subgroups: <65 vs. ≥65 years old, male vs. female, definitive vs. adjuvant radiotherapy approach, smoker vs. non-smoker, inpatients vs. outpatients and concomitant chemotherapy vs. no chemotherapy.

Abbreviation: *a: within group, p-value Kruskal-Wallis test; b: between groups p-value: Mann-Whitney U test, FU: follow up, RT: radiotherapy, SD: standard deviation, VAS: visual analog scale*

Abbreviation: *a: within group, p-value Kruskal-Wallis test; b: between groups p-value: Mann-Whitney U test, FU: follow up, RT: radiotherapy, SD: standard deviation, VAS: visual analog scale*

| **<65y vs. ≥65y  health index values** | | | | | | | |  |  |  |
| --- | --- | --- | --- | --- | --- | --- | --- | --- | --- | --- |
|  | Questionnaires (n) | Mean of <65y | SD | p-value^a^ | Questionnaires (n) | Mean of ≥65y | SD | p-value^a^ | p-value^b^ |  |
| Baseline | 105 | 0,823 | 0,146 | 0,871 | 117 | 0,782 | 0,229 | 0,277 | 0,976 |  |
| RT end | 54 | 0,830 | 0,179 |  | 59 | 0,837 | 0,177 |  | 0,885 |  |
| 1 FU | 45 | 0,836 | 0,179 |  | 44 | 0,787 | 0,228 |  | 0,481 |  |
| 2 FU | 14 | 0,844 | 0,115 |  | 18 | 0,725 | 0,274 |  | 0,423 |  |
| 3 FU | 36 | 0,870 | 0,134 |  | 18 | 0,761 | 0,318 |  | 0,850 |  |
| 4 FU | 17 | 0,832 | 0,213 |  | 5 | 0,866 | 0,104 |  | 0,986 |  |
| 5 FU | 24 | 0,899 | 0,100 |  | 9 | 0,817 | 0,174 |  | 0,327 |  |
| Total | 295 |  |  |  | 270 |  |  |  |  |  |
| **Male vs. female  health index values** | | | | | | | |  |  |  |
|  | Questionnaires (n) | Mean of male | SD | p-value^a^ | Questionnaires (n) | Mean of female | SD | p-value^a^ | p-value^b^ |  |
| Baseline | 153 | 0,822 | 0,196 | 0,541 | 68 | 0,765 | 0,231 | 0,118 | **0,034** |  |
| RT end | 77 | 0,837 | 0,150 |  | 36 | 0,816 | 0,188 |  | 0,561 |  |
| 1 FU | 62 | 0,805 | 0,206 |  | 27 | 0,828 | 0,205 |  | 0,774 |  |
| 2 FU | 26 | 0,750 | 0,235 |  | 6 | 0,872 | 0,119 |  | 0,487 |  |
| 3 FU | 37 | 0,818 | 0,236 |  | 17 | 0,868 | 0,171 |  | 0,375 |  |
| 4 FU | 15 | 0,820 | 0,225 |  | 8 | 0,883 | 0,090 |  | >0.999 |  |
| 5 FU | 21 | 0,874 | 0,133 |  | 12 | 0,882 | 0,122 |  | 0,688 |  |
| Total | 391 |  |  |  | 174 |  |  |  |  |  |
|  |  |  |  |  |  |  |  |  |  |  |
| **Definitive vs. adjvant radiotherapy** | | | | | | | | | |  |
|  | Questionnaires (n) | Mean of definitive | SD | p-value^a^ | Questionnaires (n) | Mean of adjuvant | SD | p-value^a^ | p-value^b^ |  |
| Baseline | 138 | 0,777 | 0,223 | 0,092 | 83 | 0,849 | 0,173 | 0,193 | **0,023** |  |
| RT end | 64 | 0,825 | 0,152 |  | 49 | 0,837 | 0,177 |  | 0,429 |  |
| 1 FU | 58 | 0,790 | 0,219 |  | 32 | 0,858 | 0,171 |  | 0,121 |  |
| 2 FU | 11 | 0,734 | 0,175 |  | 20 | 0,789 | 0,249 |  | **0,047** |  |
| 3 FU | 29 | 0,752 | 0,262 |  | 25 | 0,928 | 0,081 |  | **0,010** |  |
| 4 FU | 12 | 0,859 | 0,180 |  | 11 | 0,817 | 0,214 |  | 0,569 |  |
| 5 FU | 24 | 0,888 | 0,128 |  | 9 | 0,847 | 0,128 |  | 0,391 |  |
| Total | 336 |  |  |  | 229 |  |  |  |  |  |
|  |  |  |  |  |  |  |  |  |  |  |
|  |  |  |  |  |  |  |  |  |  |  |
| **Smoker vs. non-smoker** | | | | | | | | | |  |
|  | Questionnaires (n) | Mean of smoker | SD | p-value^a^ | Questionnaires (n) | Mean of non-smoker | SD | p-value^a^ | p-value^b^ |  |
| Baseline | 76 | 0,817 | 0,177 | 0,080 | 67 | 0,832 | 0,212 | 0,806 | 0,116 |  |
| RT end | 60 | 0,833 | 0,162 |  | 42 | 0,836 | 0,169 |  | 0,779 |  |
| 1 FU | 50 | 0,831 | 0,174 |  | 34 | 0,774 | 0,252 |  | 0,762 |  |
| 2 FU | 19 | 0,740 | 0,222 |  | 12 | 0,817 | 0,228 |  | 0,106 |  |
| 3 FU | 29 | 0,842 | 0,189 |  | 23 | 0,810 | 0,255 |  | 0,879 |  |
| 4 FU | 14 | 0,903 | 0,097 |  | 7 | 0,704 | 0,278 |  | 0,122 |  |
| 5 FU | 13 | 0,906 | 0,105 |  | 15 | 0,856 | 0,147 |  | 0,541 |  |
| Total | 261 |  |  |  | 200 |  |  |  |  |  |
|  |  |  |  |  |  |  |  |  |  |  |
| **Inpatient vs.outpatient** | | | | | | | | | |  |
|  | Questionnaires (n) | Mean of inpatient | SD | p-value^a^ | Questionnaires (n) | Mean of outpatient | SD | p-value^a^ | p-value^b^ |  |
| Baseline | 106 | 0,812 | 0,201 | **0,048** | 115 | 0,797 | 0,216 | 0,176 | 0,941 |  |
| RT end | 71 | 0,806 | 0,164 |  | 42 | 0,870 | 0,153 |  | **0,017** |  |
| 1 FU | 62 | 0,797 | 0,210 |  | 26 | 0,843 | 0,198 |  | 0,127 |  |
| 2 FU | 20 | 0,712 | 0,243 |  | 11 | 0,875 | 0,138 |  | **0,007** |  |
| 3 FU | 31 | 0,784 | 0,244 |  | 23 | 0,901 | 0,155 |  | **0,031** |  |
| 4 FU | 10 | 0,832 | 0,166 |  | 9 | 0,850 | 0,236 |  | 0,498 |  |
| 5 FU | 22 | 0,892 | 0,128 |  | 11 | 0,847 | 0,125 |  | 0,288 |  |
| Total | 322 |  |  |  | 237 |  |  |  |  |  |
|  |  |  |  |  |  |  |  |  |  |  |
|  |  |  |  |  |  |  |  |  |  |  |
| **Chemotherapy vs.no chemotherapy** | | | | | | | | | |  |
|  | Questionnaires (n) | Mean of chemotherapy | SD | p-value^a^ | Questionnaires (n) | Mean of no chemotherapy | SD | p-value^a^ | p-value^b^ |  |
| Baseline | 92 | 0,870 | 0,134 | **0,021** | 117 | 0,764 | 0,239 | **0,031** | 0,012 |  |
| RT end | 51 | 0,831 | 0,144 |  | 60 | 0,832 | 0,179 |  | 0,624 |  |
| 1 FU | 48 | 0,813 | 0,195 |  | 41 | 0,810 | 0,219 |  | 0,605 |  |
| 2 FU | 15 | 0,705 | 0,275 |  | 16 | 0,830 | 0,146 |  | 0,111 |  |
| 3 FU | 25 | 0,753 | 0,252 |  | 29 | 0,904 | 0,154 |  | **0,003** |  |
| 4 FU | 14 | 0,816 | 0,214 |  | 8 | 0,881 | 0,150 |  | 0,579 |  |
| 5 FU | 23 | 0,897 | 0,127 |  | 9 | 0,833 | 0,127 |  | 0,142 |  |
| Total | 268 |  |  |  | 280 |  |  |  |  |  |
|  |  |  |  |  |  |  |  |  |  |  |
|  |  |  |  |  |  |  |  |  |  |  |
|  |  |  |  |  |  |  |  |  |  |  |
