# Supplementary material for: Patient reported outcomes based on EQ-5D-5L questionnaires in head and neck cancer patients: a real-world study
Source: BMC Cancer. 2022 Nov 29;22:1236. doi: 10.1186/s12885-022-10346-4 (PMC9710161; doi:10.1186/s12885-022-10346-4)
Supplement: Supplementary file 2 — Additional file 2. [file 12885_2022_10346_MOESM2_ESM.docx]

**Supplement table 2:** Corresponding EQ-VAS values in H&N cancer patients at the baseline, the end, the first, second, third, fourth, and fifth follow up for the following subgroups: <65 vs. ≥65 years old, male vs. female, definitive vs. adjuvant radiotherapy approach, smoker vs. non-smoker, inpatients vs. outpatients and concomitant chemotherapy vs. no chemotherapy.

| **<65y vs. ≥65y  health index values** | | | | | | | |  |  |
| --- | --- | --- | --- | --- | --- | --- | --- | --- | --- |
|  | Questionnaires (n) | Mean of <65y | SD | p-value^a^ | Questionnaires (n) | Mean of ≥65y | SD | p-value^a^ | p-value^b^ |
| Baseline | 105 | 60,38 | 24,01 | **<0.0001** | 117 | 63,58 | 23,87 | 0,155 | 0,343 |
| RT end | 54 | 54,63 | 20,71 |  | 59 | 77,80 | 21,30 |  | **0,009** |
| 1 FU | 45 | 62,53 | 23,40 |  | 44 | 64,09 | 22,29 |  | 0,750 |
| 2 FU | 14 | 66,54 | 21,93 |  | 18 | 64,72 | 25,00 |  | 0,849 |
| 3 FU | 36 | 76,78 | 18,29 |  | 18 | 78,33 | 23,39 |  | 0,384 |
| 4 FU | 17 | 69,71 | 19,96 |  | 5 | 59,00 | 14,32 |  | 0,217 |
| 5 FU | 24 | 75,63 | 14,39 |  | 9 | 68,33 | 17,85 |  | 0,212 |
| Total | 295 |  |  |  | 270 |  |  |  |  |
| **Male vs. female  health index values** | | | | | | | |  |  |
|  | Questionnaires (n) | Mean of male | SD | p-value^a^ | Questionnaires (n) | Mean of female | SD | p-value^a^ | p-value^b^ |
| Baseline | 153 | 63,34 | 24,12 | **0,001** | 68 | 59,18 | 23,43 | 0,057 | 0,142 |
| RT end | 77 | 70,78 | 28,45 |  | 36 | 58,06 | 21,49 |  | 0,655 |
| 1 FU | 62 | 65,31 | 23,12 |  | 27 | 58,70 | 21,55 |  | 0,113 |
| 2 FU | 26 | 65,00 | 24,08 |  | 6 | 68,00 | 21,68 |  | 0,677 |
| 3 FU | 37 | 77,70 | 18,80 |  | 17 | 76,41 | 22,77 |  | 0,889 |
| 4 FU | 15 | 68,67 | 18,56 |  | 8 | 64,29 | 21,30 |  | 0,408 |
| 5 FU | 21 | 73,81 | 11,93 |  | 12 | 73,33 | 20,93 |  | 0,883 |
| Total | 391 |  |  |  | 174 |  |  |  |  |
| **Definitive vs. adjvant radiotherapy** | | | | | | | | | |
|  | Questionnaires (n) | Mean of definitive | SD | p-value^a^ | Questionnaires (n) | Mean of adjuvant | SD | p-value^a^ | p-value^b^ |
| Baseline | 138 | 61,26 | 24,42 | **0,010** | 83 | 63,41 | 23,19 | **0,0004** | 0,598 |
| RT end | 64 | 71,88 | 27,80 |  | 49 | 60,00 | 20,46 |  | 0,959 |
| 1 FU | 58 | 59,30 | 22,35 |  | 32 | 70,44 | 21,99 |  | **0,013** |
| 2 FU | 11 | 55,45 | 20,18 |  | 20 | 71,00 | 23,65 |  | 0,067 |
| 3 FU | 29 | 73,28 | 22,13 |  | 25 | 81,96 | 16,22 |  | 0,145 |
| 4 FU | 12 | 63,75 | 19,67 |  | 11 | 71,50 | 18,42 |  | 0,256 |
| 5 FU | 24 | 72,08 | 16,35 |  | 9 | 77,78 | 12,77 |  | 0,461 |
| Total | 336 |  |  |  | 229 |  |  |  |  |
|  |  |  |  |  |  |  |  |  |  |
| **Smoker vs. non-smoker** | | | | | | | | | |
|  | Questionnaires (n) | Mean of smoker | SD | p-value^a^ | Questionnaires (n) | Mean of non-smoker | SD | p-value^a^ | p-value^b^ |
| Baseline | 76 | 63,16 | 23,34 | **0,0005** | 67 | 65,33 | 22,21 | 0,252 | 0,637 |
| RT end | 60 | 56,83 | 21,41 |  | 42 | 63,57 | 19,04 |  | 0,187 |
| 1 FU | 50 | 61,78 | 24,07 |  | 34 | 64,26 | 20,97 |  | 0,723 |
| 2 FU | 19 | 64,21 | 23,70 |  | 12 | 67,50 | 23,79 |  | 0,833 |
| 3 FU | 29 | 79,48 | 17,75 |  | 23 | 73,87 | 23,01 |  | 0,468 |
| 4 FU | 14 | 65,71 | 13,42 |  | 7 | 68,57 | 29,11 |  | 0,671 |
| 5 FU | 13 | 67,86 | 24,86 |  | 15 | 74,67 | 14,33 |  | 0,518 |
| Total | 261 |  |  |  | 200 |  |  |  |  |
|  |  |  |  |  |  |  |  |  |  |
| **Inpatient vs.outpatient** | | | | | | | | | |
|  | Questionnaires (n) | Mean of inpatient | SD | p-value^a^ | Questionnaires (n) | Mean of outpatient | SD | p-value^a^ | p-value^b^ |
| Baseline | 106 | 63,13 | 23,36 | **0,0043** | 115 | 61,06 | 24,53 | **0,0007** | 0,452 |
| RT end | 71 | 68,10 | 12,80 |  | 42 | 64,40 | 20,49 |  | **0,048** |
| 1 FU | 62 | 61,19 | 22,06 |  | 26 | 66,54 | 27,19 |  | 0,160 |
| 2 FU | 20 | 57,48 | 25,55 |  | 11 | 75,00 | 22,47 |  | 0,069 |
| 3 FU | 31 | 70,56 | 25,43 |  | 23 | 83,43 | 13,80 |  | 0,059 |
| 4 FU | 10 | 66,54 | 19,73 |  | 9 | 68,33 | 19,20 |  | 0,936 |
| 5 FU | 22 | 74,32 | 15,91 |  | 11 | 72,27 | 15,23 |  | 0,600 |
| Total | 322 |  |  |  | 237 |  |  |  |  |
|  |  |  |  |  |  |  |  |  |  |
|  |  |  |  |  |  |  |  |  |  |
| **Chemotherapy vs.no chemotherapy** | | | | | | | | | |
|  | Questionnaires (n) | Mean of chemotherapy | SD | p-value^a^ | Questionnaires (n) | Mean of no chemotherapy | SD | p-value^a^ | p-value^b^ |
| Baseline | 92 | 66,07 | 22,12 | 0,016 | 117 | 59,74 | 24,79 | **0,031** | 0,065 |
| RT end | 51 | 72,55 | 12,7 |  | 60 | 62,83 | 19,99 |  | 0,109 |
| 1 FU | 48 | 62,9 | 21,24 |  | 41 | 63,78 | 24,64 |  | 0,584 |
| 2 FU | 15 | 62 | 24,55 |  | 16 | 68,75 | 22,55 |  | 0,434 |
| 3 FU | 25 | 69 | 23,32 |  | 29 | 84,45 | 13,08 |  | 0,008 |
| 4 FU | 14 | 52,19 | 30,95 |  | 8 | 74,38 | 13,74 |  | 0,081 |
| 5 FU | 23 | 73,91 | 16,09 |  | 9 | 74,44 | 14,88 |  | 0,907 |
| Total | 268 |  |  |  | 280 |  |  |  |  |
|  |  |  |  |  |  |  |  |  |  |
